# Construction of Recombinant Marek's Disease Virus (rMDV) Co-Expressing AIV-H9N2-NA and NDV-F Genes under Control of MDV's Own Bi-Directional Promoter

**DOI:** 10.1371/journal.pone.0090677

**Published:** 2014-03-05

**Authors:** Zhenjie Zhang, Chengtai Ma, Peng Zhao, Luntao Duan, Wenqing Chen, Fushou Zhang, Zhizhong Cui

**Affiliations:** 1 College of Veterinary Medicine, Shandong Agricultural University, Taian, China; 2 Animal Disease Prevention Technology and Research Center of Shandong Province, Taian, China; University of California, Davis, United States of America

## Abstract

To qualitatively analyze and evaluate a bi-directional promoter transcriptional function in both transient and transgenic systems, several different plasmids were constructed and recombinant MDV type 1 strain GX0101 was developed to co-express a Neuraminidase (NA) gene from Avian Influenza Virus H9N2 strain and a Fusion (F) gene from the Newcastle disease virus (NDV). The two foreign genes, NDV-F gene and AIV-NA gene, were inserted in the plasmid driven in each direction by the bi-directional promoter. To test whether the expression of pp38/pp24 heterodimers are the required activators for the expression of the foreign genes, the recombinant plasmid pPpp38-NA/1.8kb-F containing expression cassette for the two foreign genes was co-transfected with a pp38/pp24 expression plasmid, pBud-pp38-pp24, in chicken embryo fibroblast (CEF) cells. Alternatively, plasmid pPpp38-NA/1.8kb-F was transfected in GX0101-infected CEFs where the viral endogenous pp38/pp24 were expressed via virus infection. The expression of both foreign genes was activated by pp38/pp24 dimers either via virus infection, or co-expression. The CEFs transfected with pPpp38-NA/1.8kb-F alone had no expression. We chose to insert the expression cassette of Ppp38-NA/1.8kb-F in the non-essential region of GX0101ΔMeq US2 gene, and formed a new rMDV named MZC13NA/F through homologous recombination. Indirect fluorescence antibody (IFA) test, ELISA and Western blot analyses indicated that F and NA genes were expressed simultaneously under control of the bi-directional promoter, but in opposite directions. The data also indicated the activity of the promoter in the 1.8-kb mRNA transcript direction was higher than that in the direction for the pp38 gene. The expression of pp38/pp24 dimers either via co-tranfection of the pBud-pp38-pp24 plasmid, or by GX0101 virus infection were critical to activate the bi-directional promoter for expression of two foreign genes in both directions. Therefore, the confirmed function of the bi-directional promoter provides better feasibilities to insert multiple foreign genes in MDV genome based vectors.

## Introduction

Marek's disease viruses (MDV) belong to a subgroup of the alphaherpesviridae [1]. Serotype 1 MDV is the prototype virus for this group of avian viruses, it contains a double strand DNA genome of about 178kb. The genome has a unique long (U_L_) and unique short (U_S_) sequences flanked with terminal repeat long (T_RL_) and internal repeat long (I_RL_) or internal repeat short (I_RS_) and terminal repeat short (T_RS_) [2]. So far, more than 100 genes or ORFs have been identified in the MDV genome. The 1.8-kb mRNA transcript family [3], [4] and the 38kd phosphorylated protein gene (pp38) [5], [6] are two of many MDV-specific genes. They were located on I_RL_ region and separated by a short sequence of only about 305 bp but with several enhancer motifs such as TATA-box, CAAT-box, Oct-1, and Sp1, and as a bi-directional promoter to initiate transcription of two genes in opposite directions [7], [8], [9]. By use of *GFP* gene and chloramphenicol acetyltransferase (*CAT*) as reporter genes, it was demonstrated that the activity of the bi-directional promoter could be strongly increased by the expression of MDV pp38/pp24 dimers as a trans-acting transcriptional factor [10]. However, it is still unclear whether this bi-directional promoter is able to simultaneously drive two genes expression in both directions.

In the past decades, development of recombinant vaccines has been one of the most active aspects of molecular virology. Several different attenuated viruses were used as vectors to express foreign antigens from other viruses. The viruses have large genomes were more favored as vectors, such as fowl pox virus (FPV) and MDV. Successful examples have been reported in expressing viral genes such as F gene of Newcastle disease virus (NDV) [11], [12] or hemagglutinin gene of avian influenza virus (AIV) [13], [14], [15]. However, with the widely existing maternal antibodies against FPV, these recombinant FPVs were strongly denied for commercial use. In contrast to FPV, MDVs are less influenced by maternal antibodies, and a recombinant Herpesvirus of turkeys (HVT) expressing VP2 gene of infectious bursal disease virus (IBDV) has been successfully used as a commercial vaccine to control IBDV in chickens. In recent years, recombinant MDV vaccines expressing other foreign genes were studied and reported, and some rMDV vaccines have demonstrated good protective immunity in SPF or commercial chickens [16], [17], [18]. In the most studies, foreign genes were expressed under control of some common promoters such as CMV, SV40, or β-actin, which are also foreign sequences to MDV. An exception was using MDV endogenous promoter for gB gene to drive the express of NDV-F gene, and this rMDV vaccine was demonstrated as an effective and stable polyvalent vaccine against both vvMDV and NDV even in the presence of maternal antibodies [16]. In this study, we designed experiments to test if the MDV endogenous bi-directional promoter would drive the expression of two foreign genes in recombinant MDV.

Bacterial artificial clone (BAC) technology is a very powerful technique in molecular virology. With the BAC vector system, many strains MDV BAC infectious clones were constructed and used to study gene function or construction of recombinant vaccines [19], [20], [21]. Recently, we successfully constructed and rescued recombinant MDVs with a very virulent MDV field strain, GX0101, as well as its meq-deletion mutant, GX0101ΔMeq [22], [23]. Our studies indicated that GX0101Δmeq not only lost its pathogenicity but also could provide better protective immunity than the classical vaccine strain CVI988/Rispens against the challenge of very virulent MDV [24]. Similar results were also demonstrated by others in the field with a meq-deleted Md5 strain of MDV constructed based on cosmid system [25], [26], [27], [28].

**Figure 1:**
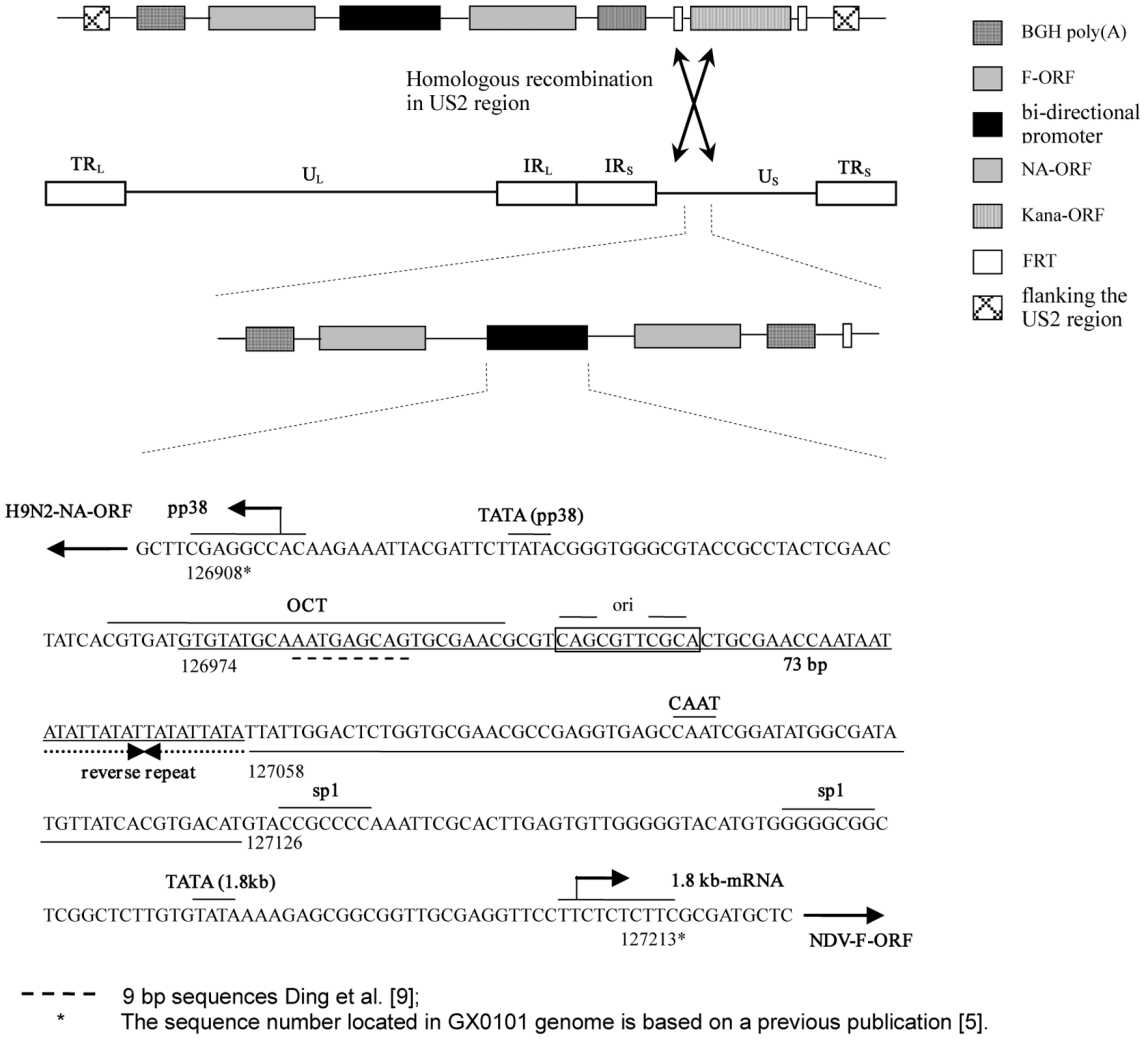
The structure of insertion sites of the H9N2-AIV-NA and NDV-F gene under the control of the bi-directional promoters on MZC13NA/F genome (GX0101/Δmeq/Kan^−^/gpt^−^/NA/F).

In this study, we attempt to insert and express the NDV-F gene and AIV-H9N2-NA gene in the GX0101Δmeq, under the control of MDV endogenous bi-directional promoter (Fig. 1). It will be more interesting to evaluate the protective immunity against MDV, NDV and AIV in the future studies.

## Materials and Methods

### Viruses and cell culture

MDV GX0101 is a field strain isolated from a layer farm in Guangxi Province of China [29]. Infectious meq-deleted BAC-GX0101 virus(GX0101/Δmeq/Kan^−^/gpt^+^) was previously constructed and rescued in our studies [22], [23]. NDV TZ060107 strain (GenBank Accession No. FJ011448) was originally isolated from a poultry farm [30]; AIV-H9N2 LG1 strain was kindly provided by Qilu Animal Health Products co (Shandong, China). All cell cultures were maintained in Dulbecco's minimum essential medium (DMEM) supplemented with 5% fetal bovine serum (FBS), 100 U/ml penicillin and 100 ug/ml streptomycin. Cells were incubated at 37°C in an atmosphere containing 5% CO_2_. All culture reagents were purchased from Gibco (Grand Island, USA).

### Antibodies and reagents

Chicken polyclonal anti-F serum raised against NDV TZ060107, mouse anti-NA polyclonal serum and mouse anti-pp38 monoclonal antibody H19 were all obtained from Avian Disease and Oncology diagnostic Laboratory (Taian, China); The pp38 and pp24 genes co-expression plasmid pBud-pp38-pp24 was constructed previously [31]; Platinum high fidelity Taq DNA polymerase, were purchased from Invitrogen (CA, USA); Gel Extraction Kit and Plasmid Maxi Kit were purchased from QIAGEN (Hilden, Germany).

### RT-PCR

RNA were extracted using Trizol reagent (TransGen Biotech, Beijing, China) as described by the manufacturer. The RT-PCR Access kit (Promega, Wisconsin, USA) was used to detect the transcription of F and NA genes. The method was essentially the same as that described previously using primer pairs #1 or #2 (Table 1) [3]. To include the pol(A) at 3′ end of expressed genes, the PCR products of NA gene (1431bp), and F gene (1692bp), were cloned directly into the pcDNA3.1(-) expressing vector (Invitrogen, California, USA) to form a new vector named as pcDNA-NA or pcDNA-F, and sequences were confirmed by automated sequencing (BGI, Beijing, China).

**Table 1:** List of primers used for construction of different recombinant plasmids.

No.	Primers	Sequence of primers(5′-3′)	Related genes and sizes of the expected PCR products
1	F-P1*	CTGGCTAGCGTTAGCATGGACCGCGCGGTTAAC	NDV-F gene (1692 bp)
	F-P2*	CTCGGTACCAAGCTATTAAACTCTATCATCCTTG	
2	NA-P1*	CCCTCTAGATCAGCATGGACCCAAATCAGAAGAT	AIV-NA gene (1431 bp)
	NA-P2*	GCAGAATTCCACCTATTATATAGGCATGAAGTTGA	
3	pp38-P1*	CGAGCGGCCGCCACCTGGCTAGCGTTGAGCATCGCG	The bi-directional promoter
		AAGAGAGA	(392 bp)
	pp38-P2*	ATGCCTGCAGGTCGGACTCTAGAGGATCCGTCGACAA	
		GGCTT CGAGGCCACAAGAAATT	
4	F-pol(A)-p1*	CTGGCTAGCGTTAGCATGGACCGCGCGGTTAAC	NDV-F gene and BGH-pol(A)
	F-pol(A)-p2*	CGAGCGGCCGCCACTGGGGATACCCCCTAGAG	(1932 bp)
5	NA-pol(A)-p1*	TCCGTCGACAAGAGCATGGACCCAAATCAGAAGAT	AIV-NA gene and BGH-pol(A)
	NA-pol(A)-p2*	GACTCTAGAGGATGGGGATACCCCCTAGAG	(1671 bp)
6	Kan^R^-F*	GACTCTAGAGGATCGTGTAGGCTGGAGCTGCTTC	Kan^R^ cassette flanked by FRT
	Kan^R^-R*	ATGCCTGCAGGTCGCATTCCGGGGATCCGTCGAC	sites (1332 bp)
7	US2-F/NA-Ka-F**	**ATGGGTGTGTCCATGATAACTATAGTCACACTTCTAG**	NA-F-Kan^R^ cassette gene with
		**ATGAATGCGATCG** CACTGGGGATACCCCCTAGAG	MDV sequence flanking the US2
	US2-F/NA-Ka-R**	**CTACTCATTTGAGGTGGTTCGATTTCCGGAGGTTTTA**	arm (5435 bp)
		**GAGGATTGGGTGG** CATTCCGGGGATCCGTCGAC	
8	US2-F	GGTTTTAGAGGATTGGGTGG	The junction between MDV-US2
	US2-R	CTTCTAGATGAATGCGATCG	and Kan-cassette (600bp)
9	F-pro-F	TGCTCACTCCTCTTGGCGAC	Labeling NDV F probes with
	F-pro-R	GCTGCATCTTCCCAACTGCC	digoxigenin (300 bp)
10	NA-pro-F	AGTTGGGTGTCCCGTTTCAT	Labeling H9N2 NA probes with
	NA-pro-R	CACTT CCTGACAATGGGCTA	digoxigenin (330 bp)
11	pp38-pro-F	GACGCGTTCGCACTGCTCATTTG	Labeling H9N2 F probes with
	pp38-pro-R	CGTTGCCGTTCGATCCAGGTCTC	digoxigenin (190 bp)

* For the convenience of plasmid construction, Restriction Endonuclease sites introduced with the primers were underlined. ** For primers US2-F/NA-Ka-F and US2-F/NA-Ka-R, underlined sequences in the upper case indicate the sequences from template constructed used to amplify co-expression cassette, and sequences in bold indicate MDV sequence flanking the US2 region.

### Plasmids construction

MDV viral DNA was prepared from the GX0101-infected CEFs as previously described [32]. To obtain the bi-directional promoter element, PCR were carried out using GX0101 DNA as the template with the primer pair #3 (Table 1). The *NotI* and *NheI* sites were introduced to the 5′ end of the pp38-P1 primer and the *XbaI* and *SalI* sites were introduced to the 5′ end of the pp38-P2 primer (underlined positions of primers, Table 1) for the convenience of plasmid construction. The 392 bp PCR product was purified with the Gel Extraction kit (Qiagen) and sequenced (BGI, Beijing, China). The recovered DNA fragment was then subcloned into pMD18-T vector to form a new transfer vector named as pBiP.

The NA or F gene, includes the pol(A) at 3′ end, were amplified with gene-specific primer pairs #4 or #5 (Table 1) from plasmid pcDNA-NA or pcDNA-F. Different constructions were designed to express NA or F separately or in combo. For individual gene expression, the NA-pol(A) fragment was inserted into the plasmid pBiP at the downstream of pp38 bi-directional promoter between the *SalI* and *XbaI* sites, result in plasmid, pPpp38-NA. Similarly, the F-pol(A) was amplified and inserted into the plasmid pBiP at the upstream of pp38 bi-directional promoter, between the *NheI* and *NotI* sites and the outcome recombinant plasmid was named pP1.8kb-F. To express both foreign genes in combo, the NA-pol(A) fragment was cloned between the *SalI* and *XbaI* sites of pP1.8kb-F resulted in plasmid pPpp38-NA/1.8kb-F. The Kan^R^ cassette flanked by FRT sites was amplified using primer pair #6 (Table 1) from pKD13 [33], then the Kan fragment was cloned between *XbaI* and *Sse8387I* sites of pPpp38-NA/1.8kb-F creating plasmid pPpp38- NA/1.8kb-F-Kan.

### Plasmid transfection

CEF cells were trypsinized, propagated from primary CEFs. About 6×10^5^ cells were plated in each well of 24-well plates with DMEM medium supplemented with 5% FCS (fetal calf serum) and free of antibiotics. Cells were incubated at 37°C for 18–24 hrs till 90–95% confluent. Plasmid transfection was performed by using LipofectAMI-NETM reagent (Gibco, BRL) according to the manufacturer's instructions. Briefly, 0.8 µg plasmid DNA and 2 µL LipofectAMI-NETM were added into two polypropylene tubes separately with 50 µL of OPTI-MEM I medium free of serum and antibiotic. Two solutions were then mixed and incubated for 20 min at room temperature and then added into another 400 µL plain DMEM. Total of 0.5 ml of transfection solution was carefully dropped onto the cell monolayers in each well. 4 hrs after incubating at 37°C CO_2_ incubator, 0.5 ml of complete medium with 5% FCS was added to each transfected cell monolayers. All plates were maintained at 37°C in a CO_2_ incubator.

When pBud-pp38-pp24 was co-transfected with pPpp38-NA, pP1.8kb-F or pPpp38-NA/1.8kb-F,0.8 µg of each plasmid DNA was used and accordingly 4 µL of LipofectAMI-NETM reagent was used to prepare the transfection mixture for each well. The transfection was performed following the steps described above.

To express pPpp38-NA, pP1.8kb-F or pPpp38-NA/1.8kb-F in GX0101-infected CEFs, primary CEFs were seeded in a 60 cm^2^ flask and inoculated with GX0101 stocks of about 1×10^5^ plaque form unit (PFU). Around 3–4 days post infection, the cell pathogenic effects (CPE) were visualized in about a quarter of cells in the monolayers. The GX0101-infected CEF monolayers were trypsinized and resuspended in DMEM medium supplemented with 5% FCS. Un-infected primary CEF cells were also trypsinized and resuspended as well. Both types of cells were then counted and then mixed (infected cells:uninfected cells = 1:2) and seeded into 24-well plate (6×10^5^ cells per well). Transfections were then carried out when the monolayers formed about 18 hrs later.

### Indirect immunofluorescence assay (IFA)

IFA assays were conducted To check the expression of genes introduced to the cells following standard procedures, Briefly, two days after transfection, cells were fixed with acetone and ethanol solution (3∶2) for 5 min at room temperature, followed by blocking with 10% normal goat serum in PBS for 30 min at room temperature. The cells were then incubated with the chicken polyclonal anti-F serum or mouse anti-NA polyclonal serum at a dilution of 1∶100 in blocking buffer for 1 hr at 37°C. Lastly, the cells were incubated with corresponding FITC-conjugated secondary antibodies, anti-chicken IgG (Fc) (1∶500 dilution with the diluent; Bethyl Laboratories, Inc., Montgomery, TX) to detect signal of F gene expression and anti-mouse IgG to detect signal of NA gene expression. Cells were washed with PBS (0.15 M NaCl, 15 mM Na_3_PO_4_, pH 7.4) twice between each step. At the end the NA and F co-expressed cells were evaluated by fluorescent microscopy.

The CEF cells in each group were trypsinized and washed 3 times with PBS, and then fixed with acetone and ethanol solution for 5 min at room temperature. The cells were suspended in 1% FBS (prepared with calcium/magnesium free PBS) to the density of about 1×10^6^ cells/ml. IFA assay was performed following the steps described above. The cell pellet was resuspended in PBS with 1% FBS for analysis on flow cytometer after centrifugation at 2000×g.

### Construction of MZC13NA/F

GX0101-BAC DNA containing the whole genome of GX0101 were transformed into Escherichia coli (*E. coli*) EL250 cells. A single clone was picked and grown in Luria-Bertani (LB) medium containing chloramphenicol (25 µg/ml) at 37°C overnight. The overnight culture was then inoculated into 10 ml of LB medium (with chloramphenicol) and grown at 32°C until an optical density at 600 nm of 0.5 was reached. The cultures were then induced to express the *recE*, *recT*, and *λ gam* proteins at 42°C, followed by chilling on ice for 15 min. The cells were then collected to be prepared as electrocompetent cells by a standard protocol [34], [35], [36]. Co-expression cassette with Kan^R^ cassette flanked by FRT sites at 3′ end of the cassette (Fig. 1) was amplified from template DNA, pPpp38-NA/1.8kb-F-Kan, using primer pair #6 (Table 1) and the PCR products were purified. About 300 ng of the PCR products were electroporated into 50 µL of electrocompetent EL250 cells harboring the GX0101-BAC using standard electroporation parameters (2.0 kV, 100 Ω and 25 µF). After electroporation, the cells were grown in 1 ml of SOC medium (2% Trypton, Oxoid; 0.5% yeast extract, Oxoid; 0.05% NaCl; 2.5 mM KCl; 10 mM MgCl_2_; 20 mM glucose) for 2 hrs and spread onto LB agar plates containing chloramphenicol (Cm, 25 µg/ml) and kanamycin (Kan, 50 µg/ml).Colonies were picked and grown in liquid LB medium with proper antibiotics. Excision of the Kan^R^ cassette was carried out by addition 0.1% arabinose (Sigma Aldrich, St. Louis, MO) into the medium to induce expression of FLPe recombinase. Then the cultures were plated on LB plates that contained Cm. After overnight incubation, colonies were picked and transferred to plates that contained either Cm or Kan. Recombinant clones, which were selected for growth on the plate with Cm, were confirmed by PCR. The EL250 cells harboring MZC13NA/F were grown up and the BAC DNA was prepared using commercially available kits (Qiagen) according to the standard protocols [37].

### The MZC13NA/F virus infected cell culture characterization

The growth kinetics and the size of vial plaques of MZC13NA/F were compared with those of wild type virus, GX0101. One-step growth kinetics of MZC13NA/F were performed as described previously [38], [39]. Briefly, for each virus MZC13NA/F or GX0101, 100 plaque-forming units (PFU) of virus were plated to infect fresh CEFs separately. At 0, 24, 48, 72, 96, 120, 144 and 170 hrs postinfection, virus-infected CEFs were harvested and a serial of 2-fold dilutions were prepared and distributed in triplicate onto the 96-well plates of CEFs. The titers of the virus at each time point were calculated based on the number of PFUs formed at each dilution and the growth curves of MZC13NA/F and GX0101 were determined.

### Southern blot hybridization

The recombinant virus MZC13NA/F was generated based on homologous recombination of plasmid DNA and wild type virus GX0101at the insertion site of US2 region, therefore the genetic purity of the MZC13NA/F clone needs to be verified. To do this, a PCR amplification targeted the US2 region with primer pair, #8 (Table 1), was performed using DNA prepared from MDV infected CEFs. The amplified PCR products were hybridized with NA or F probes. The NA or F probe DNA were amplified using primer pairs #9 or #10 (Table 1), respectively, and were labeled with digoxigenin (DIG) according to the manufacturer's manual (Boehringer Mannheim, Mannheim, Germany). The DIG-labeled hybridized DNA was detected by enzyme-linked immunoassay using anti-DIG antibodies conjugated with alkaline phosphatase and subsequent enzyme-catalyzed color reaction with 5-bromo-4-chloro-3-indolyl phosphate and nitro blue tetrazolium salt.

### NA, F and pp38 mRNA analysis

In order to quantitate the transcription level of NA, F and pp38 mRNA driven by different promoters in MZC13NA/F, a highly sensitive real-time relative quantification of reverse transcription and polymerase chain reaction (RT-qPCR) was used for quantifying the NA, F and pp38 mRNA in the infected CEF cells. The NA, F or pp38 DNA fragment was each amplified and constructed in the pMD18-T vector in order to generate the plasmid DNA standards in the quantitative assay. DNA concentration was determined by spectrophotometry at 260 nm. Serial dilutions ranging from 10^9^ copies/µL to 10^1^ copies/µL were used as standard controls. To generate standard curves, the crossing point (Cp) values in every dilution were measured in triplicates and then plotted against the logarithm of the corresponding template copy numbers. Each standard curve was generated by linear regression of the plotted points.

The primer pairs (#9, 10 and 11) and probes for the detection of NA, F and pp38 genes were designed at the highly conserved region and each amplify a fragment around 300 bp in length. The secondary CEF cells were cultured in one 6-well tissue culture plates (1×10^6^ cells each well) in order to have six repeats for each testing sample. When the CEF cells form monolayer, one 6-well tissue culture plate was inoculated with 100 PFU of MZC13NA/F and cultured for 4 days. RNA was extracted from the infected cells in every well according to the product manual of the OMEGA Viral RNA Kit (Omega Bio-Tek, Inc., Georgia, USA). Then DNA fragments were synthetized by reverse transcription with TaKaRa RNA PCR Kit (AMV) Ver.3.0 (TaKaRa, Dalian, China). 20 µl the PCR mixture was prepared according to the product manual of TaKaRa SYBR Premix ExTaqTM (TaKaRa, Dalian, China) and the data was analyzed using Applied Biosystems 7500 Fast Real-Time PCR System (ABI, Foster City, USA).

### Indirect immunofluorescence assay (IFA) on virus infected cells

About 1×10^5^ PFU of MZC13NA/F virus were inoculated in a 24-well plate of secondary CEFs and incubated for 3–4 days until CPE can be visualized. The cells were fixed with 70% acetone at room temperature. IFA assay was performed following the steps described above to confirm NA and F protein co-expression in cultured CEF cells infected with the MZC13NA/F.

### ELISA

To quantitate the expression of NA, F and MDV-pp38 protein, CEFs transfected with each transfer plasmid were harvested, washed with PBS, and added to the wells of three 96-well microtiter plates at identical cell concentrations, respectively. These plates were dried overnight at 37°C. Then, mouse anti-NA polyclonal serum (diluted 1∶200), chicken antiserum raised against NDV (diluted 1∶200) and mouse anti-pp38 monoclonal antibody H19 (diluted 1∶1,000) in PBS containing 5% fetal bovine serum was added to each well respectively, and the plates were incubated overnight at 4°C. After extensive washing of the plates with PBS containing 0.05% Tween 20, anti-mouse or chicken IgG labeled with HRP (Bio-Rad Laboratories) at a 1∶300 dilution in the same buffer was added, and incubation continued for 1 hr at 37°C. After another wash as before, the wells were developed by adding 0.1 ml of the ABTS (2,2′-azino-bis-[3-ethylbenzthiazoline-6-sulfonic acid]; Sigma Chemical Co., St. Louis, Mo.) solution (0.5 mg/ml) and incubating for 30 min at room temperature. The absorbance at 405 and 490 nm was read with a spectrophotometer.

### Western blot

Western blot analysis was carried out following the established procedures. Briefly, after 3–4 days post infection, MZC13NA/F-infected CEFs were washed twice with PBS and directed lysed with 700 ul of SDS-PAGE loading buffer (8 M urea, 10 mM Tris-HCl (pH 6.8), 2% SDS, 2% 2-mercaptoethanol, and 0.1% bromophenol blue) via boiling and vertexing. A 20 ul of the cell lysates was subjected to 10% SDS-polyacrylamide gels, followed by electrophoretically transferring to a nitrocellulose membrane of 0.45 µm pore size (Millipore, USA). The membranes were blocked with 1% bovine serum albumin (BSA) in PBS followed by washing with PBS containing 0.1% Tween-20 (PBS-T). The membranes were cut into strips to incubate with different antibodies individually at 37°C for 2 hrs. The following primary antibodies were properly diluted and applied to each strip with labeling: mouse anti-NA polyclonal antibody at a dilution of 1∶200, chicken antiserum against NDV (TZ060107) at a dilution of 1∶200 and mouse anti-pp38 monoclonal antibodies H19 at a dilution of 1∶1000. All strips of membrane were well washed and then incubated with horseradish peroxidase (HRP)-conjugated goat anti-mouse or anti-chicken IgG (Southern Biotech, USA). After washing 5 times the signals protein image were visualized by chemiluminescence with LAS-1000 (DAB) according to the manufacturer's manual.

## Results

### The expression of AIV-NA or NDV-F is dependent on the presence of pp38/pp24 dimers

**Figure 2:**
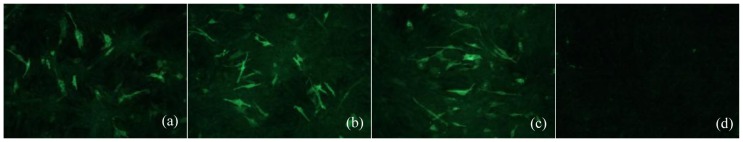
Demonstration of H9N2-NA or NDV-F expressing cells in IFA with monospecific sera in CEF transfected with different plasmids or their combinations (×200). (a) IFA with mouse anti-NA polyclonal serum to NA in CEF co-transfected with pPpp38-NA and pBud-pp38-pp24; (b) IFA with chicken polyclonal anti-F serum to F in CEF co-transfected with pP1.8kb-F and pBud-pp38-pp24; (c) IFA with mouse anti-NA polyclonal serum and chicken polyclonal anti-F serum to NA and F in CEF co-transfected with pPpp38-NA/1.8kb-F and pBud-pp38-pp24; (d) Detection of NA and F in CEF transfected with pPpp38-NA/1.8kb-F only.

**Figure 3:**
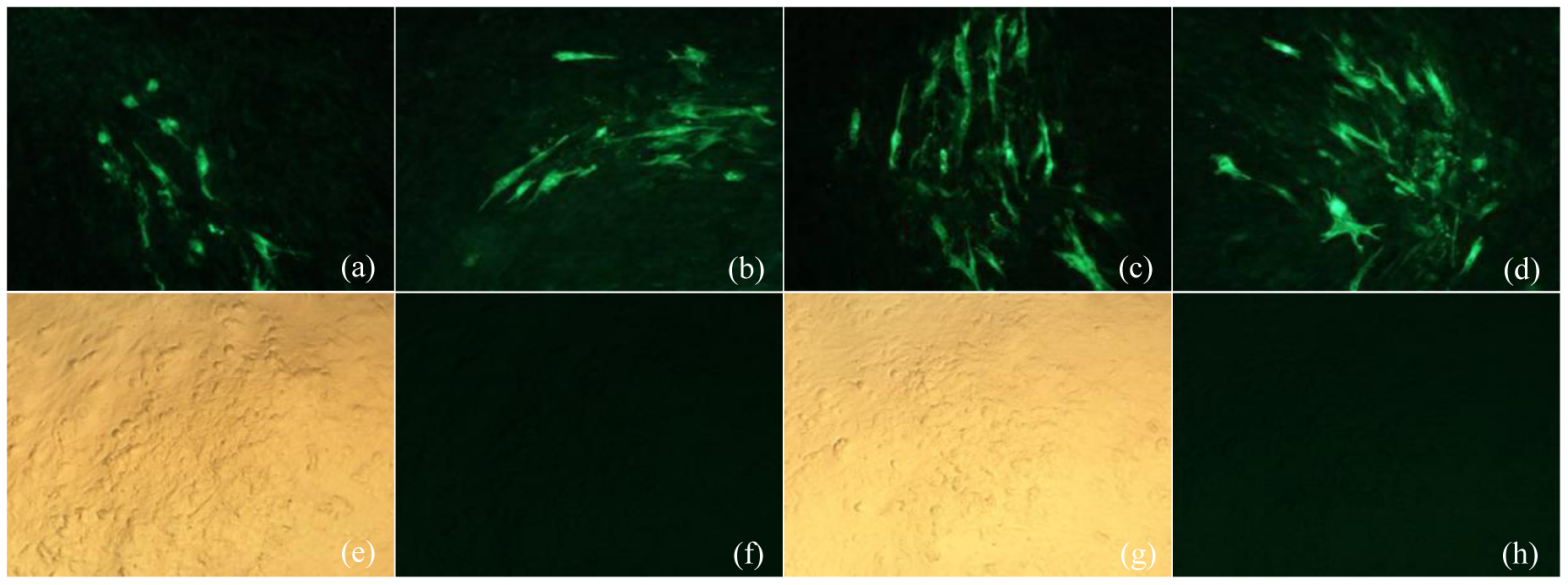
Demonstration of H9N2-NA or NDV-F expressing cells in IFA with specific antibodies in GX0101-CEF transfected with different plasmids (×200). (a) IFA with mouse anti-NA polyclonal serum to NA in GX0101-CEF transfected with pPpp38-NA; (b) IFA with chicken polyclonal anti-F serum to F in GX0101-CEF transfected with pP1.8kb-F; (c) IFA with mouse anti-NA polyclonal serum to NA in GX0101-CEF transfected with pPpp38-NA/1.8kb-F; (d) IFA with chicken polyclonal anti-F serum to F in GX0101-CEF transfected with pPpp38-NA/1.8kb-F; (e,f) IFA with mouse anti-NA polyclonal antibody against NA in GX0101-CEF; (g,h) IFA with chicken polyclonal anti-F serum against F protein in GX0101-CEF.

As it is demonstrated in Fig 2, in the presence of pp38/pp24 dimer expression in the CEF cells, the expression of AIV-NA (Fig. 2 a and c) and NDV-F(Fig. 2 b and c) genes can be detected by specific anti-sera via IFA. No NA or F protein expression was detected in CEFs transfected with pPpp38-NA/1.8kb-F only (Fig. 2 d). Expression of AIV-NA and NDV-F were also detected when the plasmid DNA, pPpp38-NA/1.8kb-F were transiently transfected with GX0101 infected CEFs, Moreover, the positive fluorescence signal were co-localized with typical MDV infection plaques (Fig. 3 a, b, c and d). These results suggested that the bi-directional promoter could co-transcript foreign genes NA and F in two directions and the pp38/pp24 dimers expressed by pBud-pp38-pp24 in co-transtected cells or endogenously by GX0101 infection were critical to activate the bi-directional promoter for expression of two foreign genes in two directions at the same time. The results were also summarized in Table 2.

**Table 2:** Comparison of the expression of NDV-F or AIV-NA in the presence or absence of pp38/pp24 dimers via IFA.

Cells	Transfected with plasmids^a^					
	pPpp38-NA	pP1.8kb-F	pPpp38-NA + pBud -pp38-pp24	pP1.8kb-F + pBud-pp38-pp24	pPpp38-NA/1.8kb-F	pPpp38-NA/1.8kb-F+ pBud-pp38-pp24
**CEF**	−	−	38%	57%	NA(−); F(−)	NA(29%); F(32%)
**GX0101-infected CEF**	65%	80%	ND^b^	ND	NA(51%); F(100%)^c^	ND

a The intensity of protein expression is based on the detection rate of positive cells by flow cytometry. When the plasmids constructed by the bi-directional promoter transfect CEF, the molar ratio of different plasmids is basically the same, which could enter the cells to minimize the influence of transcriptional activity due to the different molar ratios, while there is no correlation between the molar ratio and transcription activity of bi-directional promoter transfecting CEF in two directions.

b ND = not determined.

c It is taken the ratio of positive cells appeared in the MDV-infected CEF which was transfected with the pPpp38-NA/1.8kb-F plasmid as 100%, and the data in the table was the relative expression intensity 48 hrs post different plasmids transfection.

### Generation, Purification and Verification of MZC13NA/F

**Figure 4:**
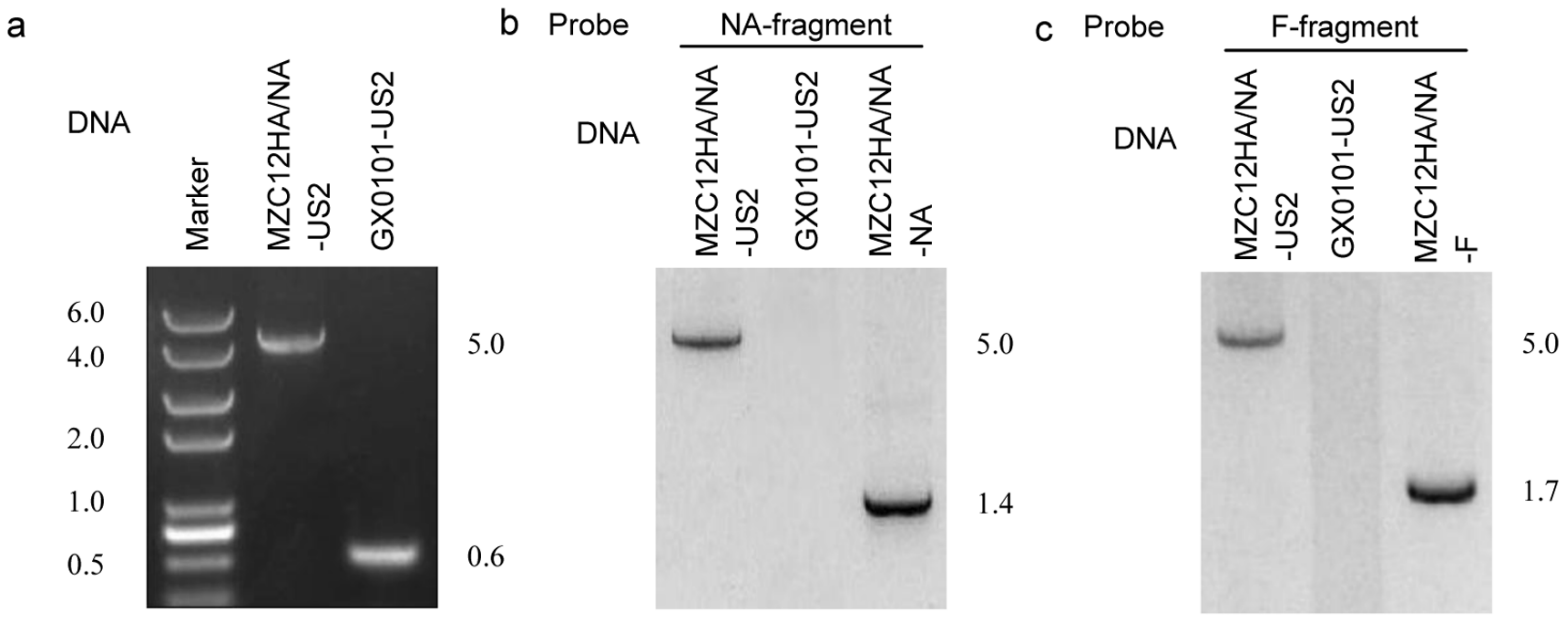
Southern blot of PCR products amplified from GX0101 and its recombinant genomes to demonstrate foreign genes. (a) Amplification of MZC13NA/F and GX0101 US2 gene by PCR. The US2 gene was amplified from DNAs prepared from the MZC13NA/F-infected CEF cells, or GX0101-infected CEF cells. The reaction products were submitted to agarose gel electrophoresis and stained with ethidium bromide; (b) The same DNAs and the NA ORF amplified from DNAs prepared from MZC13NA/F-infected CEF cells were processed for Southern blot analysis with the NA probe; (c) The same DNAs and the F ORF amplified from DNAs prepared from MZC13NA/F-infected CEF cells were processed for Southern blot analysis with the F probe.

To rescue MZC13NA/F viruses, the BAC DNA was transfected into CEF using Lipofectamine reagent according to manufacturer's instructions (Invitrogen). The transfected CEFs exhibited the CPE at around 5–6 d post transfection of MZC13NA/F BAC DNA. Highly purified MZC13NA/F was obtained after four rounds of plaque purification and proliferation. Schematic presentation of the recombinant virus genome of MZC13NA/F (Fig.1) identified the insertion site of the NA and F gene driven by the bi-directional promoters at the US2 gene. DNA prepared from MZC13NA/F infected CEFs and GX0101 virus infected CEFs were analyzed with PCR and Southern blot assays (Fig. 4 A, B and C). A 0.6 kb US2 fragment was amplified from GX0101infected cells, while the US2 fragment amplified from MZC13NA/F infected cells was about 5.0 kb, demonstrating the insertion of DNA sequences (Fig. 4 A). The PCR products were then probed with NA fragment and F fragment in southern blot, and confirmed presence of NA gene and F gene in MZC13NA/F virus infected cells, while the DNA from GX0101 virus infected cells clearly showed negative with NA and F probe(Fig. 4 B and C).

### Characterization of Recombinant MZC13NA/F

**Figure 5:**
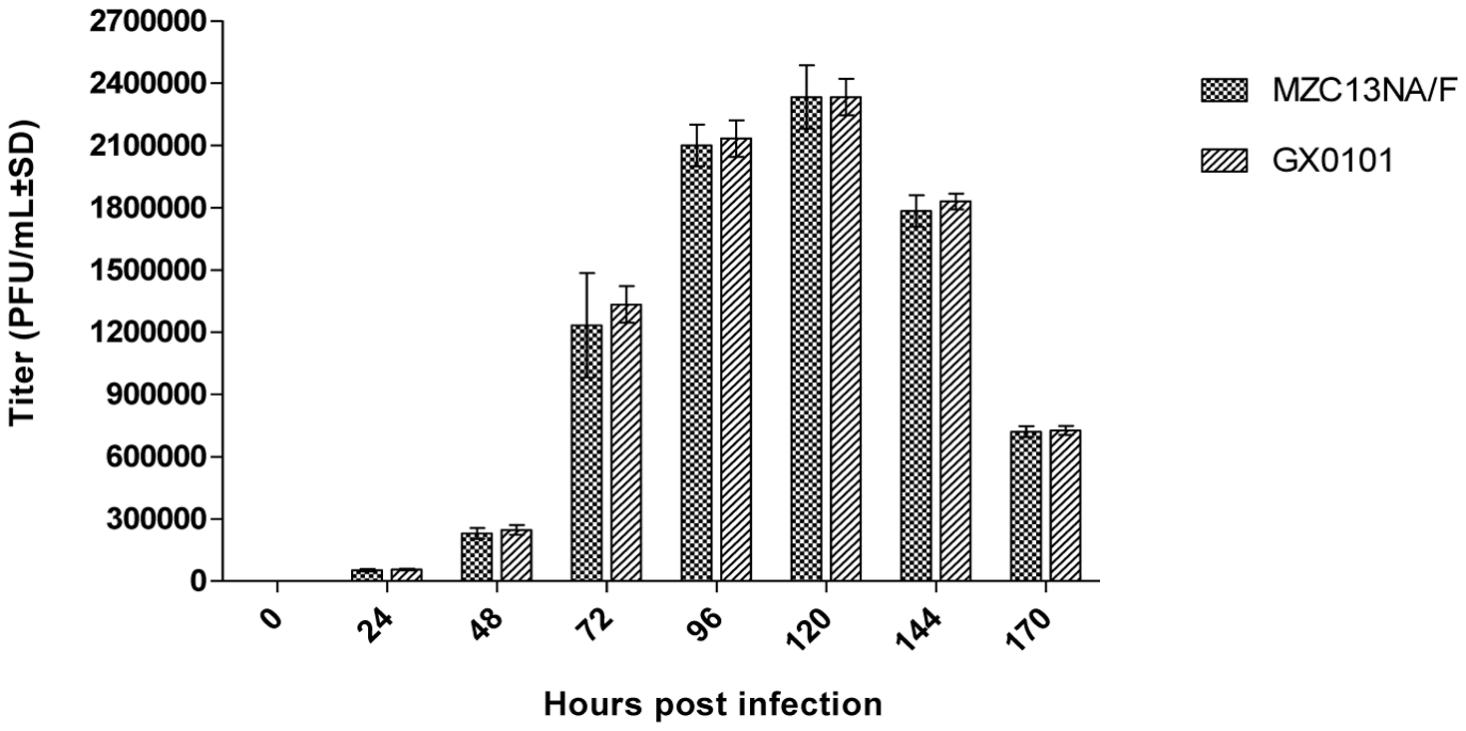
Growth curves (one-step growth kinetics) of GX0101 and its recombinant MZC13NA/F. After inoculation of CEFs with 100-infection until maximal titers were reached. The data of the growth curve of MZC13NA/F were cited from reference [40]. The results represent the mean±standard deviations of three independent replicates.

**Figure 6:**
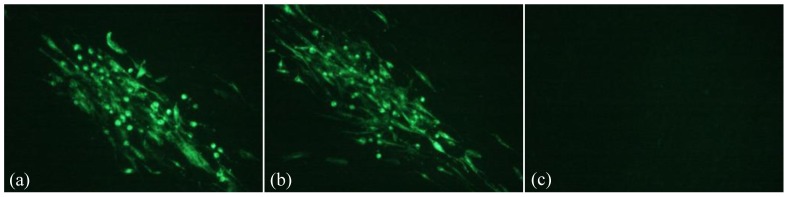
Plaque sizes of Meq-deleted MDV GX0101 and its recombinant MZC13NA/F in infected CEF cells at second passage (×200). The mouse anti-pp38 monoclonal antibody H19 bound to the MZC13NA/F (a) or its parent GX0101 (b) infected CEF cells by IFA, or to the CEF cells (c). MZC13NA/F plaque size was similar to GX0101 at some level, 96 hours post-infection showing bulging and aggregation of rounded cells.

The growth curves (one-step growth kinetics) were compared between recombinant MZC13NA/F virus and the wild type MDV1 strain GX0101. Similar growth kinetics were demonstrated between these two viruses (Fig. 5). The titers for both viruses started increasing steadily from 48 hrs post infection, both reached the highest titer at 120 hrs. The plaque size for each virus were also compared at 120 hrs post infection, and no difference was identified (Fig. 6).

### Comparison of promoter activity in real time quantification RT-PCR for NA and F mRNA in CEF infected with different rMDVs

**Figure 7:**
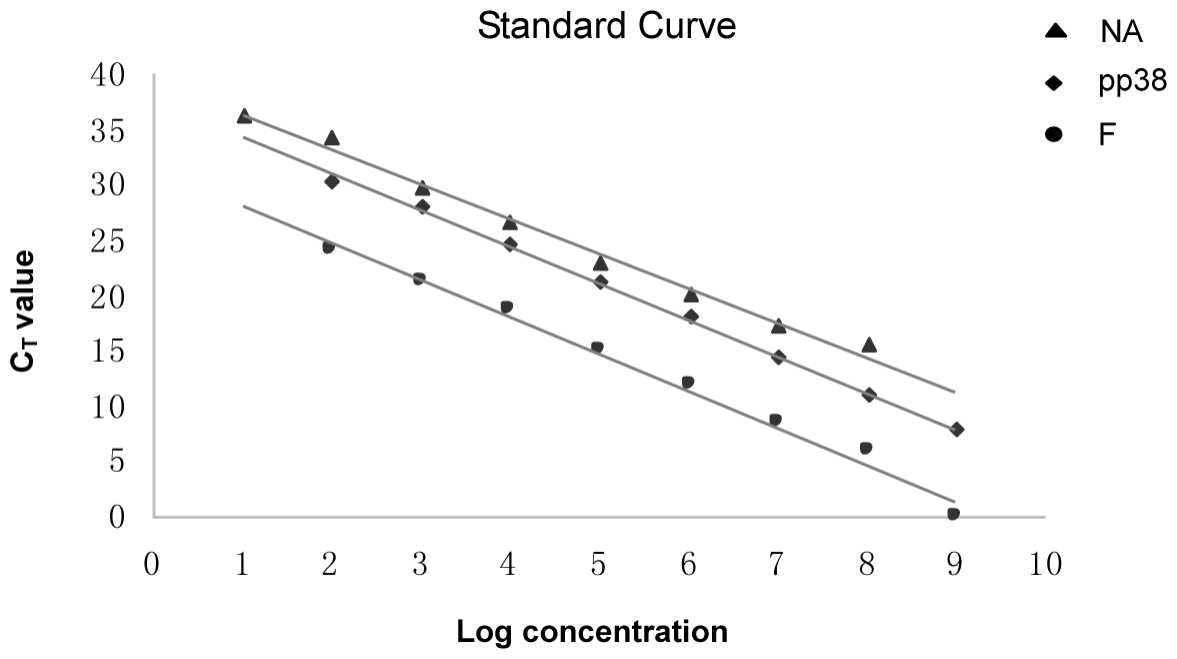
Standard curve of NA, F and pp38 assay. Copy number for the plasmid pMD18-NA, -F or -pp38 were respectively determined spectrophotometrically and diluted serially from 10^9^ copies/µL to 10^1^ copies/µL for use as standard controls. Standard curve (y  =  39.369−3.122x, y  =  31.533−3.332x and y  =  37.614−3.299x) for NA, F and pp38 genes quantification (R^2^ = 0.9918, 0.9906 and 0.9978; Efficiency = 100±1.5%) was analyzed with the GraphPad Software 5.0®. The concentration refers to the template copy number per reaction.

The RT-qPCR was done to determine the transcription efficacy of NA, F and pp38 gene driven by different promoters. In order to quantitate each cDNA, all the threshold cycle (CT) values were compared to a standard curve generated using the plasmid DNA containing the same gene of interest (Fig. 7). As shown in Table 3, the absolute quantification (AQ) value of F gene cDNA was the highest and the AQ of NA gene was the lowest. Therefore, the mRNA level of F gene in 1.8kb mRNA transcript direction was much higher than that in pp38 direction for the NA gene. The mRNA of pp38 gene was slightly higher than that of NA gene though driven by the same pp38 direction.

**Table 3:** The absolute quantification (AQ) values of cDNAs of different genes (copies/ul).

	H9N2-NA	NDV-F	MDV-pp38	Ratio of NA/F/pp38 mRNA
**RT-PCR C_T_ value Average ± standard deviation**	19.0±0.0 (6*)	21.2±0.1 (6)	20.9±0.3 (6)	−
**AQ of cDNAs**	10^5.64^	10^7^	10^5.89^	1 : 23 : 1.8

* The numbers of sample.

### Detection of Recombinant NA or F Protein Expression

**Figure 8:**
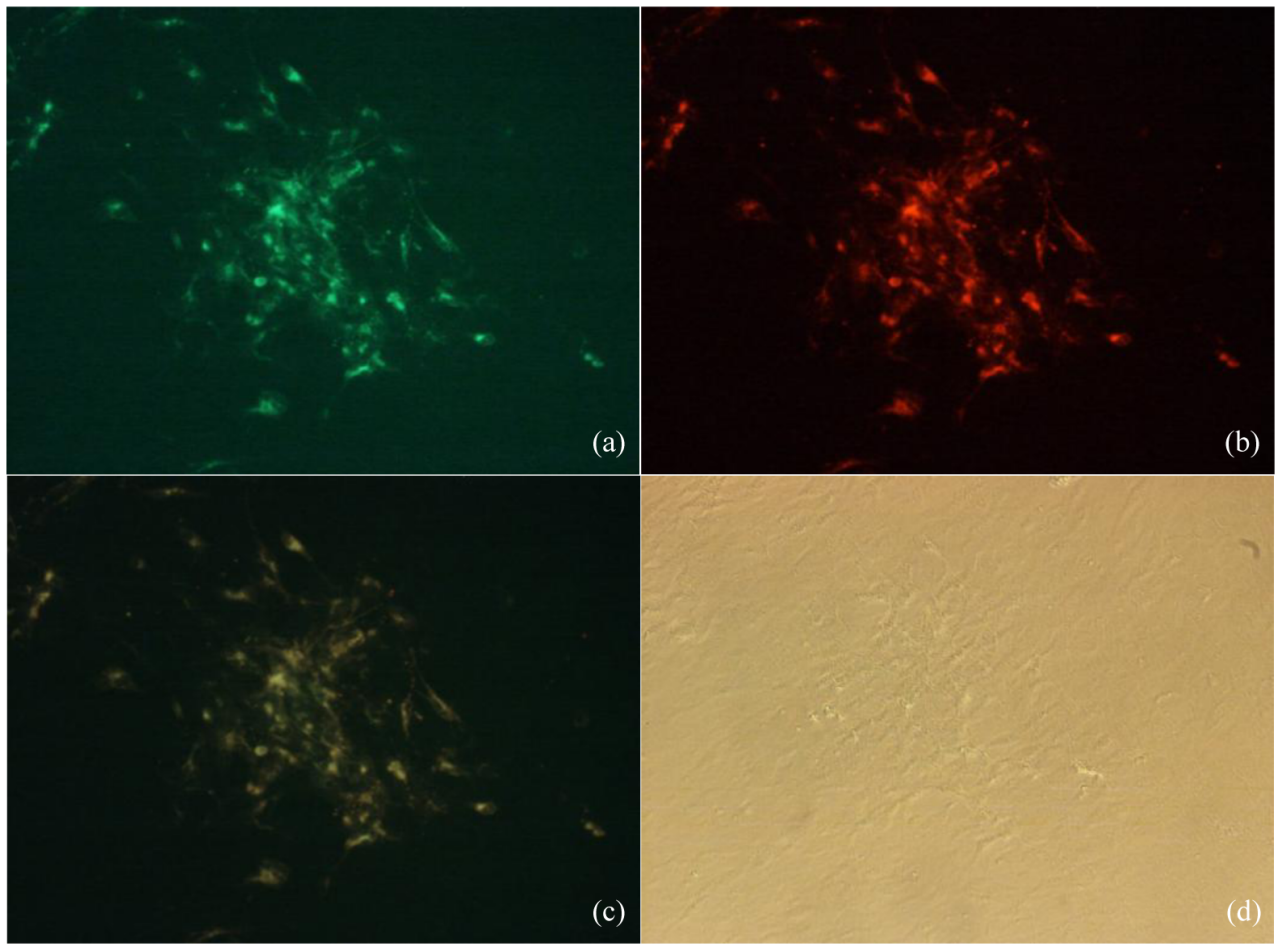
Demonstration of H9N2-NA or NDV-F expressing cells in IFA with monospecific sera in the MZC13NA/F-infected CEF (×200). (a) Distribution of NA was detected by staining with mouse anti-NA polyclonal antibody and Fluorescein Isothiocyanate-labelled anti-mouse secondary antibody (green fluorescence); (b) Distribution of F expression was detected using NDV virus-specific chicken antiserum and phycoerythrin-conjugated goat anti-chicken IgG (red fluorescence); (c) The merge including the visualization of red and green images showed that the co-expression proteins were visible in the cytoplasm or on the cell surface; (d) Photo was taken under regular light from the same plaque in the same visual field.

To confirm the expression of the recombinant NA or F proteins, CEF cells infected with purified MZC13NA/F virus were analyzed by IFA. Fluorescence signals were detected in the MZC13NA/F- infectious plaques with both mouse anti-H9 AIV-NA serum and chicken antiserum raised against NDV (Fig. 8). CEFs infected with MDV1 strain GX0101 were set as negative controls, and no detectable NA or F protein signal was observed in the cytoplasm or on the cell surface of GX0101-infected CEFs (Figure not shown).

### Comparison of the expression of F, NA and pp38 protein in MZC13NA/F-infected cells

**Figure 9:**
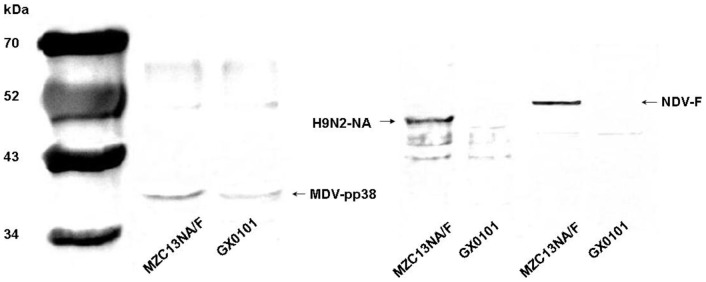
Western blot analyses of recombinant NA and F proteins co-expressed in MZC13NA/F infected CEF cells. CEF lysates prepared from the MZC13NA/F-infected CEF cells were subjected to SDS-polyacrylamide gel electrophoresis and transferred to a nitrocellulose membrane. The blotted membrane was blocked and reacted with mouse anti-pp38 monoclonal antibodies H19, mouse anti-NA polyclonal serum and chicken antiserum raised against NDV followed by HRPO-conjugated goat anti-mouse or chicken IgG antibodies and ECL Western blotting detection reagents. The prestained molecular size marker was included in the same gel and three experiments were accomplished independently.

For the NA and F genes expressed in cells infected with MZC13NA/F virus, the expression level was compared with the endogenous viral pp38 protein by ELISA assays. As presented in Table 4, the NA gene expressed under the pp38 promoter and the F gene under the 1.8kb mRNA promoter were expressed equally in cells infected with MZC13NA/F, and the level of NA or F protein expression were nearly detectable in GX0101-infected cells. It is also noticed that the expression level of pp38 protein was almost equal (1.344±0.101 vs 1.363±0.096) in MZC13NA/F or parental GX0101 infection CEF, which indicated that the introduction of bi-directional promoter did not affect the expression of viral protein pp38 itself. However, in MZC13NA/F infected cells, the foreign genes (NA or F) expressed much less than pp38 (0.445±0.031 or 1.096±0.040 vs 1.344±0.101). Even though the NA gene and pp38 gene were expressed under the same promoter in the same direction, NA was expressed much less than virus endogenous pp38 (0.445±0.031 vs 1.344±0.101). The expression of NA, F and pp38 were also demonstrated by Western Blot assay (Fig. 9).

**Table 4:** Comparison of relative expression levels of H9N2-NA and NDV-F to MDV-pp38 in MDV- GX0101 and its recombinant with H9N2-NA and NDV-F genes.

Virus	ELISA value			Ratio of NA/F/pp38
	H9N2-NA	NDV-F	MDV-pp38	
MZC13NA/F	0.445±0.031(8*)	1.096±0.040(8)	1.344±0.101(8)	1: 1.34: 3.02
GX0101	0.021±0.002(8)	0.015±0.002(8)	1.363±0.096(8)	−

* The numbers of sample.

## Discussion

Previous studies have proved the existence of a bi-directional promoter between pp38 gene and 1.8-kb mRNA transcripts of MDV genome. The sequence of this bi-directional promoter is only about 305 bp but contains several enhancer motifs [3], [5]. More *in vitro* studies from our laboratory demonstrated that the promoter could drive the expression of *GFP* or *CAT* as reporter genes in each direction with the presence of MDV pp24/pp38 hetero-dimer. The pp24/38 dimer was able to bind to the promoter and worked in-trans as a transcriptional factor to enhance the transcription activity [9], [41]. In this study, use recombinant plasmid transient transfected CEF as well as in recombinant MDV infected CEF, it was demonstrated that different foreign genes could be expressed simultaneously under control of the bi-directional promoter. As it is summarized in Table 2 and Fig.2, both AIV-NA and NDV-F genes can be expressed whether or not they were constructed in separate plasmid or in the same recombinant plasmid. Moreover NA or F gene expression in the transfected plasmid was all dependent on the presence of pp24/pp38 dimer. It is also noticed that the F gene driven by promoter p1.8kb has a higher expression level than the NA gene which is driven by promoter Ppp38. All these imply that two foreign genes can be expressed simultaneously under this bi-directional promoter in opposite directions, and the activity of the promoter in the 1.8-kb mRNA transcript direction was higher than that in the direction for the pp38 gene. The critical dependence of the pp24/pp38 dimer had been proved previously as a critical transcriptional factor to successfully express the reporter genes such as *GFP* or *CAT* driven by the bi-directional promoter [42], [43]. In this study, the pp24/pp38 dimer was either introduced by transient expression plasmid or expressed endogenously via MDV virus infection. Surprisingly, we found the foreign genes NA and F expressed much higher (Fig. 3) with the viral endogenous pp24/pp38 dimer, than transient expressed pp24/38. We speculate this was due to a high level of expression of pp24/pp38 in MDV infected cells. This may be one of the limit of this bi-birectional promoter that it would be best used in the MDV vector system, but not in other virus vectors.

Extended study were carried out by constructing recombinant MDV virus, MZC13NA/F, that contains the NA and F foreign genes driven by the same bi-directional promoter. As shown in Fig. 6 that both NDV-F and H9N2 AIV-NA antigens were successfully expressed and detected in IFA with monoclonal antibody against NDV-F or AIV-NA proteins. The results clearly indicated that the MDV endogenous bi-directional promoter of only 305 bp could effectively drive two foreign genes expressed in two directions. As mentioned above, the bi-directional promoter is located between pp38 and 1.8kb mRNA family transcription start sites and pp38/pp24 dimer could work as trans-acting transcriptional factor to strongly enhance its promoter activity [8], [9], [41], the bi-directional promoter may have some advantages in expression of two foreign genes in the early stage of MDV infection. This is because pp38 is an early gene related to MDV early B-lymphocyte cytolytic infection [44], and pp38/pp24 dimers should be available in the early stage of infection. Such suggestion is to be further confirmed in comparative studies with other common promoters such as CMV, SV40, even MDV gB promoter.

With the cosmid vector system, meq-deleted Md5 strain of MDV was constructed and confirmed to provide better protective immunity than CVI988/Rispens in chickens against very virulent plus MDV challenges [26], [27], [28]. We constructed a BAC-vectored infectious clone of very virulent MDV strain GX0101 [22], a series of meq-deleted GX0101 mutants were constructed and some of them demonstrated better protective immunity against MDV in SPF chickens than CVI988/Rispens vaccine as well [24], [45]. One of protective Meq-deleted mutant virus is SC9-1, which was fully attenuated and able to provide better protective immunity than CVI988/Rispens [45]. In fact, In this study, the recombinant MDV virus, MZC13NA/F, was derived from SC9-1 virus by inserting NDV-F and H9N2 AIV-NA expression cassette under control of MDV's own bi-directional promoter. It will be more interesting to investigate whether protective efficacy can be demonstrated when challenged with Avian Influenza virus or Newcastle Disease virus. These investigation are currently ongoing in the lab.

In construction of recombinant MDV as vectors to express foreign genes, two of the key questions are where to insert and which promoter to choose. Should a strong promoter result in higher protein expression, and will thereafter provide better protective efficacy? So far, heterogenous promoters such as CMV and SV40 promoters or homogenous promoter such as MDV gB promoter were used to drive expression of foreign genes [16], [17], [46]. In one study, the NDV-F gene was expressed in rMDV either driven by the MDV gB promoter, or driven by non-MDV promoters, such as SV40 and β-actin promoters. However, with MDV gB promoter, rMDV-F induced lower antibody titers based on ELISA assay, but it provided better protective efficacy then the F gene driven by SV40 or β-actin promoters [16]. The bi-directional promoter used in this study was also a MDV's own promoter as gB promoter, but it had unique characteristics different from gB promoter. It was able to drive two genes express simultaneously in opposite direction and its activity was strongly depended on a trans-acting transcriptional factor pp38/pp24 dimer [8].

Experiment results in both recombinant expression plasmid-transfected CEF and recombinant MZC13NA/F-infected CEF suggest that the MDV's own bi-directional promoter may have some potential use in construction of recombinant MDV vaccines expressing foreign genes of other viruses, especially for construction of recombinant MDV vaccine expressing two foreign genes. By using the bi-directional promoter, recombinant expression cassettes for two genes could be inserted into suitable sites of MDV genomes in one step, and it helps to simplify the procedure and reduce troublesome and difficulties to insert different expression cassette plasmid DNA into MDV genomes again. The strong dependence on pp38/pp24 dimer as a trans-acting transcriptional factor may have some limitation in the use of the bi-directional promoter in construction of recombinant viruses other than use MDV as viral vectors.

## References

[1] McGeoch DJ, Dolan A, Ralph AC (2000) Toward a comprehensive phylogeny for mammalian and avian herpesviruses. Journal of Virology 74: 10401–10406. 11044084 10.1128/jvi.74.22.10401-10406.2000 PMC110914

[2] Su S, Cui N, Cui ZZ, Zhao P, Li YP, et al (2012) Complete Genome Sequence of a Recombinant Marek's Disease Virus Field Strain with One Reticuloendotheliosis Virus Long Terminal Repeat Insert. Journal of Virology 86(24): 13818. 23166235 10.1128/JVI.02583-12 PMC3503109

[3] Barlic-Maganja D, Grom J (2001) Highly sensitive one-tube RT-PCR and microplate hybridisation assay for the detection and for the discrimination of classical swine fever virus from other pestiviruses. Journal of virological methods 95: 101–110. 11377717 10.1016/s0166-0934(01)00302-0

[4] Bradley G, Hayashi M, Lancz G, Tanaka A, Nonoyama M (1989) Structure of the Marek's disease virus BamHI-H gene family: Genes of putative importance for tumor induction. Journal of Virology 63: 2534–2542. 2542569 10.1128/jvi.63.6.2534-2542.1989 PMC250719

[5] Bradley G, Lancz G, Tanaka A, Nonoyama M (1989) Loss of Marek's disease virus tumorigenicity is associated with truncation of RNAs transcribed within BamHI-H. Journal of Virology 63: 4129–4135. 2550661 10.1128/jvi.63.10.4129-4135.1989 PMC251026

[6] Cui ZZ, Lee LF, Liu JL, Kung HJ (1991) Structural analysis and transcriptional mapping of the Marek's disease virus gene encoding pp38, an antigen associated with transformed cells. Journal of Virology 65: 6509–6515. 1658357 10.1128/jvi.65.12.6509-6515.1991 PMC250698

[7] Cui ZZ, Qin AJ, Lee LF (1992) Expression and processing of Marek' s disease virus pp38 gene insert cells and immunological characteriation of the gene product. Proceedings of 19th World' s Poulty Congress: 123–126.

[8] Ding JB, Cui ZZ, Jiang SJ, Sun AJ, Sun SH (2005) Study on the characterization of the bi-directional promoter between pp38 gene and 1.8kb mRNA transcripts of Marek's disease viruses. Acta Microbidogica Sinica 45(3): 363–367. 15989227

[9] Ding JB, Cui ZZ, Jiang SJ, Reddy SJ (2006) The enhancement effect of pp38 gene product on the activity of its upstream bi-directional promoter in Marek's disease virus. Science in China: Series C Life Sciences 49: 1–10. 16544576 10.1007/s11427-004-0119-y

[10] Ding JB, Cui ZZ, Lee LF (2007) Marek's disease virus unique genes pp38 and pp24 are essential for transactivating the bi-directional promoters for the 1.8-kb mRNA transcripts. Virus Genes 35(3): 643–650. 17619133 10.1007/s11262-007-0129-5

[11] Taylor J, Edbauer C, Rey-Senelonge A, Bouquet JF, Norton E, et al (1990) Newcastle disease virus fusion protein expressed in a fowl pox virus recombinant confers protection in chickens. Journal of Virology 64(4): 1441–1450. 2157037 10.1128/jvi.64.4.1441-1450.1990 PMC249277

[12] Morgan RW, Gelb J, Schreurs CS, Lütticken D, Rosenberger JK, et al (1992) Protection of chickens from Newcastle and Mark's disease with a recombinant herpserivus of turkey's vaccine expressing the Newcastl e disease virus fusion protein. Avian Dis 36: 858–870. 1485872

[13] Webster RG, Kawaoka Y, Taylor J, Weinberg R, Paoletti E (1991) Efficacy of nucleo protein and haemagglutinin antigens expressed in fowl pox virus as vaccine for influenza in chickens. Vaccine 9: 303–308. 1651609 10.1016/0264-410x(91)90055-b

[14] Swayne DE, Garcia M, Beck JR, Kinney N, Suarez DL (2000) Protection against diverse highly pathogenic H5 avian influenza viruses in chickens immunized with recombinant fowl pox vaccine containing an H5 avian influenza hemagglutinin gene insert. Vaccine 18: 1088–1095. 10590330 10.1016/s0264-410x(99)00369-2

[15] Ma MX, Jin NY, Wang ZG, Wang RL, Fei DL, et al (2006) Construction and immunogenicity of recombinant fowlpox vaccines coexpressing HA of AIV H5N1 and chicken IL18. Vaccine 24(20): 4304–431. 16621199 10.1016/j.vaccine.2006.03.006

[16] Sonoda K, Sakaguchi M, Okamura H, Yokogawa K, Tokunaga E, et al (2000) Development of an Effective Polyvalent Vaccine against both Marek's and Newcastle Diseases Based on Recombinant Marek's Disease Virus Type 1 in Commercial Chickens with Maternal Antibodies. Journal of Virology 74(7): 3217–3226. 10708438 10.1128/jvi.74.7.3217-3226.2000 PMC111822

[17] Tsukamoto K, Kojima C, Komori Y, Tanimura N, Mase M, et al (1999) Protection of Chickens against Very Virulent Infectious Bursal Disease Virus (IBDV) and Marek's Disease Virus (MDV) with a Recombinant MDV Expressing IBDV VP2. Virology 257: 352–362. 10329546 10.1006/viro.1999.9641

[18] Sakaguchi M, Nakamura H, Sonoda K, Okamura H, Yokogawa K, et al (1998) Protection of chickens with or without maternal antibodies against both Marek's and Newcastle diseases by one-time vaccination with recombinant vaccine of Marek's disease virus type 1. Vaccine 16(5): 472–479. 9491501 10.1016/s0264-410x(97)80001-1

[19] Petherbridge L, Brown AC, Baigent SJ, Howes K, Sacco MA, et al (2004) Oncogenicity of virulent Marek's disease virus cloned as bacterial artificial chromo-somes. J Virol 78: 13376–13380. 15542691 10.1128/JVI.78.23.13376-13380.2004 PMC525015

[20] Baigent SJ, Petherbridge LJ, Smith LP, Zhao Y, Chesters PM, et al (2006) Herpesvirus of turkey reconstituted from bacterial artificial chromosome clones induces protection against Marek's disease. J Gen Virol 87: 769–776. 16528024 10.1099/vir.0.81498-0

[21] Cui HY, Wang YF, Shi XM, Tong GZ, Lan DS, et al (2008) Construction of Marek's disease virus serotype 814 strain as an infectioous bacterial artificial chromosome. Chinese journal of biotechnology 24: 569–575. 18616164 10.1016/s1872-2075(08)60028-x

[22] Sun AJ, Petherbridge LP, Zhao Y, Li YP, Cui ZZ, et al (2009) A BAC clone of MDV strain GX0101 with REV-LTR integration retained its pathogenicity. Chinese Science Bulletin 54 (15): 2641–2647.

[23] Li YP, Sun AJ, Su S, Zhao P, Cui ZZ, et al.. (2011) Deletion of the Meq gene significantly decreases immunosuppression in chickens caused by pathogenic Marek's disease virus. Virology journal: 2–8. 10.1186/1743-422X-8-2 PMC3024286 21205328

[24] Su S, Li YP, Sun AJ, Zhao P, Cui ZZ, et al (2010) Protective immunity of a meqΔdeleted Marek's disease virus against very virulent virus challenge in chickens. Acta Microbiologica Sinica 50(3): 380–386. 20499644

[25] Reddy SM, Lupiani B, Gimeno IM, Silva RF, Lee LF, et al (2002) Rescue of a pathogenic Marek's disease virus with overlapping cosmid DNAs: use of a pp38 mutant to validate the technology for the study of gene function. Proc Natl Acad Sci USA 99(May (10)): 7054–7059. 10.1073/pnas.092152699 PMC124527 11997455

[26] Lee LF, Kreager KS, Arango J, Paraguassu A, Beckman B, et al (2010) Comparative evaluation of vaccine dfficacy of recombinant Marek's disease virus vaccine lacking Meq oncogene in commercial chickens. Vaccine 28(5): 1294–1299. 19941987 10.1016/j.vaccine.2009.11.022

[27] Lee LF, Zhang HM, Heidari M, Lupiani B, Reddy SM (2011) Evaluation of factors affecting vaccine dfficacy of recombinant Marek's disease virus lacking the Meq oncogene in chickens. Avian Dis.55 2: 172–179. 21793430 10.1637/9575-101510-Reg.1

[28] Lee LF, Heidari M, Zhang HM, Lupiani B, Reddy SM, et al (2012) Cell culture attenuation eliminates rMd5Meq-induced bursal and thymic atrophy and renders the mutant virus as an effective and safe vaccine against Marek's disease. Vaccine 30(34): 5151–5158. 22687760 10.1016/j.vaccine.2012.05.043

[29] Zhang Z, Cui ZZ (2005) Isolation of recombinant field strains of Marek's disease virus integration with reticuloendotheliosis virus genome fragments. Sci. China C. Life Sci 48 (1): 81–88. 10.1360/03yc0270 15844360

[30] Sun SH, Cui ZZ (2007) Biological Identification of Newcastle Disease Viruses Isolated from Eggs of a Parent Breeder Farm. Acta Veterinaria et Zootechnica Sinica 38(7): 741–743.

[31] Jiang SJ, Ding JB, Meng SS, Cui ZZ, Yang HC (2005) Co-expression and Construction of Eukaryote Plasmid of pp38 and pp24 of Marekcs Disease Virus.Virologica sinica. 20(4): 404–407.

[32] Morgan RW, Cantello JL, McDermott CH (1990) Transfection of chicken embryo fibroblasts with Marek's disease virus DNA. Avian Diseases 34: 345–351. 2164390

[33] Datsenko KA, Wanner BL (2000) One-step inactivation of chromosomal genes in Escherichia coli K-12 using PCR products. Proc Natl Acad Sci U S A 97 (12): 6640–6645. 10.1073/pnas.120163297 PMC18686 10829079

[34] Muyrers JP, Zhang Y, Testa G, Stewart AF (1999) Rapid modification of bacterial artificial chromosomes by ET-recombination. Nucleic Acids Res 27 (6): 1555–1557. 10.1093/nar/27.6.1555 PMC148353 10037821

[35] Narayanan K, Williamson R, Zhang Y, Stewart AF, Ioannou PA (1999) Efficient and precise engineering of a 200 kb beta-globin human bacterial artificial chromosome in E. coli DH10B using an inducible homologous recombination system. Gene Ther 6: 442–447. 10435094 10.1038/sj.gt.3300901

[36] Yu D, Ellis HM, Lee EC, Jenkins NA, Copeland NG, et al (2000) An efficient recombination system for chromosome engineering in Escherichia coli. Proc Natl Acad Sci U S A 97: 5978–5983. 10811905 10.1073/pnas.100127597 PMC18544

[37] Sun AJ, Xu XY, Lawrence PB, Zhao YG, Cui ZZ (2010) Functional evaluation of the role of reticuloendotheliosis virus long terminal repeat (LTR) integrated into the genome of a field strain of Marek's disease virus. Virology 397: 270–276. 19962172 10.1016/j.virol.2009.11.017

[38] Cui HY, Wang YF, Shi XM, An TQ, Tong GZ, et al (2009) Construction of an infectious Marek's disease virus bacterial artificial chromosome and characterizationof protection induced in chickens. Journal of Virological Methods 156: 66–72. 19026690 10.1016/j.jviromet.2008.10.021

[39] Schumacher D, Tischer BK, Fuchs W, Osterrieder N (2000) Reconstitution of Marek's disease virus serotype 1 (MDV-1) from DNA cloned as a bacterial artificial chromosome and characterization of a glycoprotein B-negative MDV-1 mutant. Journal of Virology 74: 11088–11098. 11070004 10.1128/jvi.74.23.11088-11098.2000 PMC113189

[40] Li Y, Reddy K, Reid SM, Cox WJ, Brown IH, et al (2011) Recombinant herpesvirus of turkeys as a vector-based vaccine against highly pathogenic H7N1 avian influenza and Marek's disease. Vaccine 29: 8257–8266. 21907750 10.1016/j.vaccine.2011.08.115

[41] Ding JB, Cui ZZ, Jiang SJ, Li YP (2008) The structure of unique genes pp38 and pp24 and the effect on the activity of its upstream bi-directional promoters in Marek's disease virus. Science in China: Series C Life Sciences 38: 760–765.

[42] Shigekane H, Kawaguchi Y, Shirakata M, Sakaguchi M, Hirai K (1999) The bi-directional transcriptional promoters for the latency-relating transcripts of the pp38/pp24 mRNAs and the 1.8 kb-mRNA in the long inverted repeats of Marek's disease virus serotype 1 DNA are regulated by common promoter-specific enhancers. Archives of Virology 144: 1893–1907. 10550664 10.1007/s007050050713

[43] Ding JB, Cui ZZ, Lee LF, Cui XP, Reddy SM (2006) The role of pp38 in regulation of Marek's disease virus bi-directional promoter between pp38 and 1.8-kb mRNA. Virus Genes 32(2): 193–201. 16604452 10.1007/s11262-005-6876-2

[44] Gimeno IM, Witter RL, Hunt HD, Reddy SM, Lee LF, et al (2005) The pp38 gene of Marek's disease virus (MDV) is necessary for cytolytic infection of B cells and maintenance of the transformed state but not for cytolytic infection of the feather follicle epithelium and horizontal spread of MDV. Journal of Virology 79(7): 4545–4549. 15767457 10.1128/JVI.79.7.4545-4549.2005 PMC1061578

[45] Su S (2013) Comparisons of Biological Characteristic between Recombinant Marek's disease virus and its Mutant Strains. Tai'an: Shandong Agricultural university.

[46] Cui HY, Gao HB, Cui XL, Zhao Y, Wang YF, et al (2013) Avirulent Marek's Disease Virus Type 1 Strain 814 Vectored Vaccine Expressing Avian Influenza (AI) Virus H5 Haemagglutinin Induced Better Protection Than Turkey Herpesvirus Vectored AI Vaccine. PLoS ONE 8(1): e53340. 23301062 10.1371/journal.pone.0053340 PMC3536743

